# Impact of obesity-related genes in Spanish population

**DOI:** 10.1186/1471-2156-14-111

**Published:** 2013-11-23

**Authors:** Fernando Martínez-García, María L Mansego, Gemma Rojo-Martínez, Griselda De Marco-Solar, Sonsoles Morcillo, Federico Soriguer, Josep Redón, Monica Pineda Alonso, Juan C Martín-Escudero, Richard S Cooper, Felipe J Chaves

**Affiliations:** 1 Hypertension Clinic, Hospital Clínico Universitario and INCLIVA, University of Valencia, Valencia 46010, Spain; 2 CIBER 03/06 Physiopathology of Obesity and Nutrition, Institute of Health Carlos III, Minister of Health, Santiago de Compostela 28071, Spain; 3 Genotyping and Genetic Diagnosis Unit, Hospital Clínico Research Foundation and INCLIVA, Valencia 46010, Spain; 4 CIBER of Diabetes and Associated Metabolic Diseases (CIBERDEM), Barcelona 08036, Spain; 5 Endocrinology and nutrition service, Carlos Haya Universitary Hospital, Málaga 29009, Spain; 6 Internal Medicine, Rio Hortega Hospital, University of Valladolid, Valladolid 47010, Spain; 7 Department of Preventive Medicine and Epidemiology, Loyola University Medical School, Maywood, IL, USA; 8 Internal Medicine, Hospital Clínico, Avda Blasco Ibañez, 17, Valencia 46010, Spain

**Keywords:** Obesity, Genetics, *FTO* gene, Genetic score

## Abstract

**Background:**

The objective was to investigate the association between BMI and single nucleotide polymorphisms previously identified of obesity-related genes in two Spanish populations. Forty SNPs in 23 obesity-related genes were evaluated in a rural population characterized by a high prevalence of obesity (869 subjects, mean age 46 yr, 62% women, 36% obese) and in an urban population (1425 subjects, mean age 54 yr, 50% women, 19% obese). Genotyping was assessed by using SNPlex and PLINK for the association analysis.

**Results:**

Polymorphisms of the *FTO* were significantly associated with BMI, in the rural population (beta 0.87, p-value <0.001). None of the other SNPs showed significant association after Bonferroni correction in the two populations or in the pooled analysis. A weighted genetic risk score (wGRS) was constructed using the risk alleles of the Tag-SNPs with a positive Beta parameter in both populations. From the first to the fifth quintile of the score, the BMI increased 0.45 kg/m^2^ in Hortega and 2.0 kg/m^2^ in Pizarra. Overall, the obesity predictive value was low (less than 1%).

**Conclusion:**

The risk associated with polymorphisms is low and the overall effect on BMI or obesity prediction is minimal. A weighted genetic risk score based on genes mainly acting through central nervous system mechanisms was associated with BMI but it yields minimal clinical prediction for the obesity risk in the general population.

## Background

Obesity is a global pandemic and a major health concern because of the consequent morbidity and premature mortality. Changes in lifestyles resulting in energy intake and expenditure imbalance have led to an increase in obesity prevalence all over the world. Although this trend is driven by the “obesogenic” environment, evidence demonstrated that it is facilitated by genetic susceptibility, being the heritability in familial and twin studies of around 40-70%, [1,2].

Although many genetic variants have consistently been associated with obesity, the individual impact on body weight seems to be small. Using genome-wide association studies (GWAS), several genes have been associated with obesity, especially the fat mass and obesity associated gene (*FTO*). Initially found in a GWAS of type 2 diabetes, *FTO* has been consistently associated with obesity and BMI in many studies [3-6]. Moreover, a published meta-analysis confirmed the association in 32387 individuals of European ancestry from 15 cohorts [7]. According to this study, the top hit within the intron 1 of *FTO* gene was the *rs1421085* although all the SNPs in high linkage disequilibrium (LD) with this variant were also strongly associated with BMI, including the *rs9939609* the most replicated that increase 31% the risk of obesity [4].

Other candidate genes have also been linked with obesity with varying degrees of association. The melanocortin 4 receptor (*MC4R*) gene, linked with monogenic obesity [8], also has a polygenic effect with the minor allele of the *c.307G > A* (*rs2229616*, *p.Val103Ile*) being protective against obesity [9,10]. Other loci, including the *INSIG2*, *TMEM18, KCTD15, SH2B1, MTCH2*, *GNPDA2, BDNF,* or *CHST8* genes*,* have also been associated with obesity. Furthermore, two other loci, one close to the *NEGR1* gene and another near *STK33* were also associated to obesity in some studies and the *NEGR1* gene has been independently associated with obesity in a pediatric cohort [11].

A genetic risk score constructed with the *FTO*, *MC4R* and six of the newly discovered loci performed poorly as a predictor of obesity and the explained variance of BMI was less than 1% [7]. Due to the heterogeneity of the trait, however, the predictive value may vary with the characteristics of the population studied. The objective of the present study was to assess the association between individual genetic variants and haplotypes of several obesity-related genes (*FTO*, *MC4R, MTCH2, NEGR1, SEC16B, INSIG2*, *TMEM18, KCTD15, SH2B1, FAIM2*, *ATXN2L, BDNF, BDNFOS*, *GNPDA2, ADRB2, PRL, PTER, ADIPOQ, ETV5, MAF, NPC1, CTNNBL1,* and *HTR2C)* and BMI in two Spanish populations. We analyzed interactions between the associated genetic variants in order to identify functional relationships between genes and metabolic pathways for BMI regulation. The potential predictive value of a weighted genetic risk score to predict obesity was also evaluated.

## Methods

Two adult general population samples were used in the present study, one from Pizarra, a village of 6600 inhabitants which is located in the south of Spain, and the other from an urban area of Valladolid located in the center of Spain. All the participants provided written informed consent and the local ethical committees approved the studies.

The Pizarra study is a population-based survey of cardiovascular risk factors. The characteristics of this population have been previously published [12,13]. Briefly, 2090 subjects aged 18-65 years were randomly selected from the municipal register. For 1119 out of 2090 individuals demographic, anthropometric and DNA were available. Persons with severe clinical or psychological problems, pregnant women and those who were institutionalized were excluded. This population has a prevalence of obesity higher than that reported for other Spanish communities. From this population, we were only able to use 869 subjects for the final analysis (131 were excluded because of lack of complete information and 119 due to that they did not pass the quality thresholds).

The selection process of the Hortega study has also been previously published [14,15]. Briefly, subjects older than 18 years old were randomly selected from the public register of the western medical area of Valladolid (Spanish National Statistical Institute; http://ine.es). Subjects were invited to participate in the study by phone. Individuals with serious concomitant diseases or psychiatric disorders were excluded. A second list of subjects was selected to replace those who reclined to take part in the study. The percentage of replacement was 32%. The calculated minimal sample size required to be representative of the population was 1400, and 1504 individuals were finally recruited. From this population, we were only able to use 1425 subjects for the final analysis (2 were excluded because of technical reasons and 77 due to that they did not pass the quality thresholds).

The names of the institutional boards which approved the study were: Institutional board of the Clinical Hospital Río Hortega in Valladolid. Institutional board of the Carlos Haya Hospital in Malaga.

### Anthropometric measurements

The anthropometric parameters were measured for all the individuals according to standard procedures in the two studies. Weight was assessed with a precise scale while the subjects were wearing light clothes and barefoot. Height was assessed in a similar way. Body mass index (BMI) was calculated as weight divided by height^2^ expressed in kg/m^2^. Obesity was defined as a BMI ≥ 30 kg/m^2^ and overweight as a BMI of 25.0–29.9 kg/m^2^[16]. Blood pressure was assessed with an automatic device following the recommendations of the European Society of Hypertension. Fasting blood samples for blood count and serum biochemistry were analyzed by an auto-analyzer.

### Type 2 diabetes definition

In Hortega subjects were considered as diabetics if they were already diagnosed of type 2 diabetes by a physician or if the plasma glucose remained equal or higher than 126 mg/dl after the extraction of a second sample in fasting conditions in those subjects with glucose equal or higher than 140 mg/dl in non fasting conditions [17].

In Pizarra, the WHO 1998 criteria were used to classify the people with diabetes, IGT and IFG [12,18]. People were also considered to have diabetes if they were already receiving treatment with oral anti-diabetics. Those people being treated with diet only received an OGTT to verify the diagnosis.

### Genotyping

Blood for the genotyping was taken into tubes with 15% of ethylenediaminetetraacetic acid (EDTA) and was kept at 4ºC to process in 5 days. Those samples which were not processed in five days after the extraction were frozen at -80ºC. DNA was isolated from peripheral blood cells using Realpure Genomic DNA extraction kit (Real Pure, Paterna, Spain) and the samples were diluted to a final concentration of 100 ng/μl. SNPs were selected based on a search in the PubMed database of previous reports about association with obesity in GWAS studies between the years 2007-2009. Selected SNPs were genotyped using an oligo-ligation assay (SNPlex; Applied Biosystems, Foster City, CA) following the manufacturer’s instructions. The characteristics of the selected SNPs and related genes are shown in Table 1. In both populations the minor allele frequencies (MAF) were quite similar to the MAF in the HapMap CEU samples. One SNP, *rs7561317,* close to the *TMEM18* gene, was excluded because of being monomorphic.

**Table 1:** Characteristics of the selected SNPs

**Locus**	**GENE name**	**HGN**	**SNP**	**TAG-SNP**	**CHR position**	**HGVS names**	**HORTEGA GEN%**	**PIZARRA GEN%**	**HORTEGA MAF**	**PIZARRA MAF**	**HWE p-value (All/controls)**
1p31.1	*NEURONAL GROWTH REGULATOR 1*	*NEGR1*	*rs3101336*		72523773	*NT_032977.8:g.42723104C > T*	96.80	100	0.237	0.252	0.472/0.663
			*rs2568958*		72537704	*NT_032977.8:g.42737035A > G*	98.86	100	0.115	0.095	0.858/0.303
			*rs2815752*		72585028	*NT_032977.8:g.42784359A > G*	98.93	100	0.355	0.349	0.929/0.304
1q25.2	*SEC16 HOMOLOG B (S. CEREVISIAE)*	*SEC16B*	*rs10913469*		176180142	*NM_033127.2:c.1881 + 177A > G*	97.80	100	0.154	0.143	0.293/1
2q14.1	*INSULIN INDUCED GENE 2*	*INSIG2*	*rs7566605*		118552495	*NT_022135.15:g.7543947C > G*	98.20	99.77	0.454	0.439	0.706/0.643
2p25.3	*TRANSMEMBRANE PROTEIN 18*	*TMEM18*	*rs2867125*		612827	*NT_022327.14:g.612827T > C*	97.47	100	0.351	0.397	0.078/0.186
			*rs6548238*		624905	*NT_022327.14:g.624905T > C*	98.93	99.88	0.449	0.419	0.043/0.141
			*rs4854344*		628144	*NT_022327.14:g.628144G > T*	98.73	99.88	0.380	0.387	0.0521/0.164
3q27	*ADIPONECTIN, C1Q AND COLLAGEN DOMAIN*	*ADIPOQ*	*rs17300539*		188042154	*NT_005612.15:g.93054610G > A*	98.53	99.54	0.433	0.403	0.032/0.342
			*rs3774261*	1	188054253	*NM_004797.2:c.215-414A > G*	98.20	99.54	0.496	0.484	0.967/0.726
3q28	*ETS VARIANT 5*	*ETV5*	*rs7647305*		187316984		95	98.85	0.118	0.125	0.902/0.655
4p13	*GLUCOSAMINE-6-PHOSPHATE DEAMINASE 2*	*GNPDA2*	*rs10938397*		44877284	*NT_006238.10:g.4884493A > G*	97.80	100	0.195	0.198	0.382/0.340
5q31-q32	*ADRENERGIC, BETA-2-, RECEPTOR, SURFACE*	*ADRB2*	*rs12654778*	1	148185934	*NT_029289.10:g.9368677G > A*	98.13	100	0.363	0.330	0.207/0.318
6p22.2-p21.3	*PROLACTIN*	*PRL*	*rs4712652*		22186594	*NT_007592.14:g.12936866G > A*	97.07	97.58	0.346	0.375	0.804/0.516
10p12	*PHOSPHOTRIESTERASE RELATED*	*PTER*	*rs10508503*		16339957	*NT_077569.2:g.10662847C > T*	96.27	99.19	0.346	0.375	0.006/0.015
11p13	*BRAIN-DERIVED NEUROTROPHIC FACTOR*	*BDNF*	*rs4923461*		27613486	*NR_002832.1:c.244-4482G > A*	98.33	99.88	0.169	0.172	0.955/0.637
			*rs925946*		27623778	*NR_002832.1:c.404 + 5650G > T*	98.47	100	0.347	0.375	0.0001/0.003
			*rs10501087*		27626684	*NR_002832.1:c.404 + 8556G > T*	98.00	99.65	0.424	0.384	0.779/0.634
			*rs6265*		27636492	*NM_001709.3:c.196G > A (p.V66M)*	98.13	100	0.461	0.489	0.373/0.426
11p14.1	*BDNF OPPOSITE STRAND (NON-PROTEIN CODING)*	*BDNFOS*	*rs4074134*		27603861	*NR_002832.1:c.244-14107G > C*	98.86	99.54	0.185	0.160	0.956/0.841
11p11.2	*MITOCHONDRIAL CARRIER HOMOLOG 2*	*MTCH2*	*rs4752856*	1	47604618	*NM_014342.2:c.681 + 590C > T*	98.40	99.77	0.240	0.261	0.928/0.699
			*rs10838738*		47619625	*NM_014342.2:c.87 + 882T > C*	97.87	99.65	0.489	0.455	1/0.545
12q13	*FAS APOPTOTIC INHIBITORY MOLECULE 2*	*FAIM2*	*rs7138803*		48533735	*NT_029419.11:g.12390774G > A*	98.66	99.42	0.354	0.344	0.391/0.111
16p11	*ATAXIN 2-LIKE*	*ATXN2L*	*rs8049439*		28745016	*NM_007245.2:c.466-46T > C*	96.53	100	0.327	0.356	0.619/0.512
16q12.2	*FAT MASS AND OBESITY ASSOCIATED*	*FTO*	*rs6499640*		52327178	*NM_001080432.1:c.45 + 31536A > G*	98.07	99.88	0.173	0.173	0.694/0.488
			*rs1421085*	1	52358455	*NM_001080432.1:c.46-43098T > C*	97.87	100	0.237	0.261	1/0.719
			*rs1121980*	1	52366748	*NM_001080432.1:c.46-34805G > A*	98.93	100	0.073	0.092	0.617/0.389
			*rs8050136*	1	52373776	*NM_001080432.1:c.46-27777C > A*	98.86	99.19	0.220	0.228	0.420/0.470
			*rs3751812*		52375961	*NM_001080432.1:c.46-25592G > T*	98.80	100	0.381	0.376	0.471/0.471
			*rs9939609*	1	52378028	*NM_001080432.1:c.46-23525T > A*	98.33	99.88	0.174	0.170	0.966/0.876
			*rs7190492*		52386253	*NM_001080432.1:c.46-15300G > A*	98.73	99.65	0.351	0.351	0.782/0.438
			*rs8044769*		52396636	*NM_001080432.1:c.46-4917C > T*	97.33	100	0.339	0.329	0.967/0.880
16q22-q23	*V-MAF MUSCULOAPONEUROTIC FIBROSARCOMA*	*MAF*	*rs1424233*		78240252		99.13	99.77	0.321	0.321	0.805/0.52
16p11.2	*SH2B ADAPTOR PROTEIN 1*	*SH2B1*	*rs4788102*		28780899	*NT_010393.15:g.20186477G > A*	98.27	99.77	0.224	0.204	0.890/0.466
18q22	*MELANOCORTIN 4 RECEPTOR*	*MC4R*	*rs17782313*		56002077	*NT_025028.13:g.5641943T > C*	98.66	100	0.455	0.464	0.215/0.382
18q11-q12	*NIEMANN-PICK DISEASE, TYPE C1*	*NPC1*	*rs1805081*		19394430	*NM_000271.3:c.644A > G (p.H215R)*	98.93	99.65	0.343	0.376	0.012/0.017
19q13.11	*POTASSIUM CHANNEL TETRAMERISATION*	*KCTD15*	*rs11084753*		39013977	*NT_011109.15:g.6590355A > G*	97.93	99.65	0.428	0.390	0.184/0.336
20q11.23-q12	*CATENIN, BETA LIKE 1*	*CTNNBL1*	*rs6013029*		35832994	*NM_030877.3:c.750 + 3134G > T*	98.27	99.88	0.267	0.283	0.574/0.482
Xq24	*5-HYDROXYTRYPTAMINE RECEPTOR 2C*	*HTR2C*	*rs3813929*		113724776	*NT_028405.11:g.250852C > T*	98.47	99.88	0.434	0.399	0.168/0.292

*HGN*: Human genome nomenclature; *SNP*: single nucleotide polymorphism; *CHR position*: chromosomal position; *HGVS*: human genome variation society; *Gen%*: genotyping call rate; *MAF*: minor allele frequency.

### Statistical analysis

Quantitative variables are expressed as the mean ± standard deviation (SD). Qualitative variables are expressed as entire counts or percentage. We used the free software Quanto v1.2.4 to calculate the statistical power with the continuous and qualitative traits under an additive genetic model taking into account the minor allele frequency of the selected SNPs, the sample size, the mean and standard deviation of BMI in our sample and the magnitude of the association for that variants in the literature with a type I error of 0.05.

To assess the association between genotypes/haplotypes and BMI, we used linear regression models adjusted by age and gender under the additive inheritance genetic model. Because of the relationship of BMI with type 2 diabetes, we also performed the analysis including type 2 diabetes as co-variable. The interaction between genetic variants and BMI was made by adding a multiplicative term within the linear regression model. The Bonferroni correction adjusted for 26 independent tests (see Additional file 1: Figure S1 about the LD patterns) was used to correct for multiple comparisons, being the corrected p-value for significance of 0.00192.

The r2 was used to measure the linkage disequilibrium (LD). Haplotype frequencies were estimated by the Expectation Maximization Algorithm (EM). Tag-SNPs, LD and haploblocks were calculated using Haploview version 3.32. The individual SNP and haplotype analysis was performed with the program PLINK v.1.06 developed by Purcell (http://pngu.mgh.harvard.edu/purcell/plink/). The average genotyping call rate to filter individuals and SNPs was 95%. We also filter those SNPs with a MAF lower than 1% and with a HWE p-value lower than 0.001. A weighted genetic risk score (wGRS) was constructed using the risk alleles of the Tag-SNPs which showed a positive Beta parameter in Hortega, Pizarra and the pooled analysis. For each individual, the number of risk alleles (0,1,2) per SNP was weighted for their effect sizes and re-scaled by dividing by the average of the all the effect sizes. The weighted risk alleles for the selected SNPs were summed for each individual, and the overall individual sum was rounded to the nearest integer to represent the individual’s risk allele score. The comparison of BMI among the quintiles of the score adjusted for age and sex and the 95% confidence interval for the means were calculated from the linear regression estimates. We used the area under the curve (AUC) from the receiver operating characteristic (ROC) curves to assess the capability of the score to predict obesity in the two populations. These procedures were performed with StataIC 11 (StataCorp4905 Lakeway drive, College Station, Texas, 77845, USA).

Finally, the statistical heterogeneity of the results for the two populations was analysed using the p-value for Cochrane’s Q statistic and the I2 heterogeneity index. This meta-analysis was also performed with PLINK considering both fixed and random effect models. Two SNPs, rs4712652 and rs1424233, were excluded from the meta-analysis because allele mismatch.

## Results

The general characteristics of the individuals from the two populations after removal of subjects with low genotyping call rate are shown in Table 2. Subjects from Pizarra population were significantly younger and had higher BMI and greater prevalence of obesity than those from the Hortega study. The gender distribution was balanced in the Hortega population whereas in the Pizarra study there were more women than men.

**Table 2:** Characteristics of the individuals included in the study for Hortega and Pizarra populations after removing those individuals with low genotyping call rate

**Variables**	**HORTEGA study (N = 1425)**	**PIZARRA study (N = 869)**
Age (years)	54.4 ± 19.3	46.2 ± 13.8***
Gender, M(%)/F(%)	718 (50.4) / 707 (49.6)	322 (37.2) / 543 (62.8)***
Weight (kg)	70.8 ± 12.9	74.0 ± 14.1***
Height (m)	1.63 ± 0.1	1.6 ± 0.1***
Waist perimeter (cm) (M/F)	95.70 ± 10.15 /83.4 ±12.8	100.6 ± 10.6/ 97.8 ±14.7***
BMI (kg/m2)	26.4 ± 4.2	28.6 ± 5.2***
SBP (mmHg)	130.8 ± 21.5	129.1 ± 21.8
DBP (mmHg)	79.3 ± 10.7	77.7 ± 12.4***
Total Cholesterol (mg/dl)	201.6 ± 38.2	202.6 ± 39.7
LDL Cholesterol (mg/dl)^¶^	114.3 ± 34.5	124.2 ± 34.1***
HDL Cholesterol (mg/dl)	51.6 ± 4.2	58.5 ± 13.2***
Triglycerides (mg/dl)	178.1 ± 114.4	99.5 ± 67.0***
Glucose (mg/dl)	92.6 ± 20.7	106.7 ± 30.3***
Obesity (N/%)	253/18. 5	307/36.0 ***
Overweight (N/%)	574/41.9	306/ 35.9*
Abdominal obesity (N/%)^+^	388/28.6	514/60.1 ***
DM2 (N/%)	109/7.7	145/18.9 ***
HTN (N/%)	602/42.3	365/42.8

*p-values denote differences between Hortega study and Pizarra study (p < 0.05); <0.01; ***p-values <0.001; *M*: Male, *F*: Female, *BMI*: Body mass index, *SBP*: Systolic blood pressure, *DBP*: Diastolic blood pressure, *DM2*: Type 2 Diabetes Mellitus, *HTN*: Hypertension; ^¶^Calculated with the Friedwald formula; ^+^Abdominal obesity was defined as waist >102 cm in men and >88 cm in women.

### Association analysis with BMI

*Pizarra population:* From 988 individuals with complete information, 119 subjects were excluded because of low genotyping rate. The genotyping call rate for the remaining individuals was 99.7 ± 0.9%. Except for the *rs7561317,* the rest of SNPs passed the thresholds for HWE, MAF or call rate. The SNP genotyping call rate for the remaining SNPs was 99.7 ± 0.45%. The results of the genetic association study in Pizarra are presented in Table 3.

**Table 3:** Individual TagSNP association analysis with BMI adjusted by age and gender under an additive genetic model in Pizarra, Hortega and the pooled analysis

							**BMI**			
**Gene**	**SNP**	**A1**	**MAF**	**Population**	**Prior statistical power**	N*	**Beta**	**Standard error**	STAT**	**P**
*FTO*	rs9939609*	*A*	**0.398**	**PIZARRA**	**32.77**	**848**	**0.875**	**0.23**	**3.79**	**0.0001573**
			0.435	HORTEGA	66.65	1345	0.1348	0.15	0.90	0.368
			0.420	POOLED	77.88	2215	0.2976	0.13	2.19	0.0287
	*rs8044769*	*T*	0.463	PIZARRA	30.96	848	−0.582	0.23	−2.54	0.0112
			0.455	HORTEGA	62.61	1331	−0.1019	0.15	−0.68	0.495
			0.458	POOLED	73.98	2198	−0.257	0.13	−1.90	0.0571
	*rs7190492*	*A*	0.328	PIZARRA	5.06	845	−0.559	0.23	−2.34	0.0192
			0.337	HORTEGA	5.16	1346	0.2065	0.16	1.29	0.195
			0.333	POOLED	5.21	2213	−0.07336	0.14	−0.51	0.6076
	*rs6499640*	*G*	0.376	PIZARRA	5.01	847	0.336	0.23	1.43	0.1511
			0.381	HORTEGA	5.02	1347	0.09312	0.15	0.60	0.543
			0.379	POOLED	5.02	2216	0.2335	0.13	1.69	0.0911
*MC4R*	rs17782313*	*C*	0.199	PIZARRA	9.69	848	0.575	0.28	1.98	0.0478
			0.191	HORTEGA	16.33	1348	0.1167	0.19	0.60	0.544
			0.195	POOLED	20.23	2218	0.3111	0.17	1.80	0.0716
*MTCH2*	*rs10838738*	*G*	0.352	PIZARRA	5.80	845	0.453	0.24	1.88	0.0603
			0.353	HORTEGA	6.98	1348	0.0972	0.15	0.62	0.536
			0.353	POOLED	7.61	2215	0.1698	0.14	1.19	0.2318
*NEGR1*	*rs3101336*	*A*	0.375	PIZARRA	7.89	848	−0.084	0.24	−0.34	0.7277
			0.343	HORTEGA	11.89	1348	−0.403	0.15	−2.64	0.0083
			0.355	POOLED	14.23	2210	−0.1586	0.14	−1.13	0.2561
*ATXN2L*	*rs8049439*	*C*	0.375	PIZARRA	8.87	848	−0.139	0.23	−0.58	0.5576
			0.338	HORTEGA	14.15	1344	0.3292	0.15	2.13	0.0331
			0.353	POOLED	17.35	2209	0.1715	0.14	1.22	0.2198
*HTR2C*	*rs3813929*	*T*	0.157	PIZARRA	10.16	847	−0.406	0.35	−1.16	0.2455
			0.182	HORTEGA	19.46	1342	−0.4365	0.23	−1.89	0.0577
			0.172	POOLED	23.29	2211	−0.5551	0.20	−2.68	0.0073
*ADRB2*	*rs12654778*	*A*	0.388	PIZARRA	3 5.35	848	0.271	0.23	1.16	0.2452
			0.380	HORTEGA	69.22	1347	−0.1452	0.15	−0.95	0.339
			0.382	POOLED	80.65	2217	0.01463	0.13	0.10	0.9152
*SEC16B*	*rs10913469*	*C*	0.142	PIZARRA	9.33	848	0.392	0.32	1.21	0.2266
			0.152	HORTEGA	16.43	1347	−0.1609	0.20	−0.78	0.432
			0.148	POOLED	19.78	2216	0.00939	0.18	0.05	0.96
*TMEM18*	*rs4854344*	*G*	0.172	PIZARRA	15.26	847	−0.198	0.30	−0.64	0.5173
			0.169	HORTEGA	30.08	1348	−0.3012	0.20	−1.48	0.138
			0.170	POOLED	37.86	2217	−0.243	0.18	−1.33	0.1815
*INSIG2*	*rs7566605*	*C*	0.327	PIZARRA	13.68	846	0.101	0.24	0.41	0.676
			0.321	HORTEGA	26.42	1348	−0.0301	0.16	−0.18	0.8514
			0.322	POOLED	33.12	2216	0.1152	0.14	0.79	0.4265
*ADIPOQ*	*rs17300539*	*A*	0.126	PIZARRA	7.59	844	−0.072	0.33	−0.21	0.8298
			0.118	HORTEGA	11.08	1348	0.1146	0.22	0.51	0.6082
			0.120	POOLED	13.15	2214	0.0818	0.20	0.40	0.6838
	*rs3774261*	*A*	0.495	PIZARRA	10.96	844	−0.036	0.22	−0.16	0.8727
			0.461	HORTEGA	19.84	1346	−0.0549	0.15	−0.36	0.7138
			0.473	POOLED	26.42	2210	0.0609	0.13	0.45	0.6503
*ETV5*	*rs7647305*	*T*	0.205	PIZARRA	10.76	838	0.022	0.28	0.079	0.9364
			0.215	HORTEGA	19.82	1325	0.2402	0.18	1.30	0.1927
			0.213	POOLED	24.39	2175	0.2106	0.16	1.26	0.2077
*GNPDA2*	*rs10938397*	*G*	0.440	PIZARRA	11.60	848	−0.338	0.22	−1.52	0.1278
			0.455	HORTEGA	21.56	1348	−0.048	0.15	−0.32	0.7492
			0.449	POOLED	26.80	2218	−0.1687	0.13	−1.26	0.2078
*PRL*	*rs4712652*	*A*	0.453	PIZARRA	5.28	828	0.203	0.23	0.87	0.3846
		*G*	0.487	HORTEGA	5.69	1326	0.0367	0.15	0.24	0.8056
		*A*	0.489	POOLED	5.91	2175	−0.115	0.13	−0.84	0.3976
*PTER*	*rs10508503*	*T*	0.093	PIZARRA	5.10	841	−0.119	0.39	−0.30	0.7617
			0.112	HORTEGA	5.27	1315	−0.279	0.23	−1.21	0.2238
			0.105	POOLED	5.34	2174	−0.3695	0.21	−1.71	0.0866
*BDNF*	*rs925946*	*T*	0.282	PIZARRA	10.40	848	0.128	0.24	0.51	0.6046
			0.266	HORTEGA	17.97	1348	−0.071	0.16	−0.44	0.6578
			0.272	POOLED	22.37	2215	0.0795	0.14	0.54	0.5855
	*rs10501087*	*C*	0.253	PIZARRA	10.01	845	0.033	0.26	0.12	0.8988
			0.231	HORTEGA	16.77	1348	0.0875	0.17	0.49	0.6231
			0.240	POOLED	20.96	2214	0.1472	0.15	0.93	0.3493
*FAIM2*	*rs7138803*	*A*	0.352	PIZARRA	7.38	844	0.155	0.24	0.65	0.5159
			0.352	HORTEGA	10.92	1348	−0.0643	0.15	−0.42	0.675
			0.351	POOLED	12.81	2214	0.0513	0.14	0.36	0.7129
*MAF*	*rs1424233*	*A*	0.483	PIZARRA	5.29	846	−0.192	0.23	−0.83	0.4074
		*G*	0.499	HORTEGA	5.70	1348	0.2336	0.14	1.57	0.1158
		*A*	0.495	POOLED	5.92	2216	−0.2422	0.13	−1.79	0.0725
*NPC1*	*rs1805081*	*G*	0.329	PIZARRA	5.25	845	−0.014	0.24	−0.05	0.9553
			0.352	HORTEGA	5.64	1345	0.0046	0.15	0.03	0.9758
			0.344	POOLED	5.83	2212	−0.0463	0.14	−0.32	0.7432
*KCTD15*	*rs11084753*	*A*	0.395	PIZARRA	5.62	846	0.093	0.23	0.39	0.6897
			0.352	HORTEGA	6.44	1343	−0.1562	0.15	−1.01	0.31
			0.367	POOLED	6.94	2210	0.0573	0.13	0.41	0.6781
*CTNNBL1*	*rs6013029*	*T*	0.091	PIZARRA	5.79	847	0.014	0.39	0.036	0.9708
			0.066	HORTEGA	6.44	1348	−0.1522	0.30	−0.50	0.6133
			0.076	POOLED	7.16	2216	0.1139	0.25	0.44	0.6547

A1: minor allele, *Number of non-missing individuals included in the analysis (top) and frequency of the haplotype in the sample (bottom); **t statistic coefficient; p-values are not corrected for multiple testing; Bold type indicates significant association after Bonferroni correction. **The SNPs, *rs4788102* in the SH2B1 and rs4074134 of the BDNFOS genes are not included because are in high LD with the SNPs, *rs8049439* of the *ATXN2L* gene (r2 0.94) and rs10501087of the BDNF gene (r2 0.95), respectively.

All SNPs of the *FTO* gene within a block of high LD in intron 1 (r2 > 0.8) were significantly associated with BMI (beta 0.87, p-value <0.001, for the most associated SNP, *rs9939609*). Their impact on BMI, based on the confident intervals for the beta parameter ranged from 0.31 to 1.34 (average 0.83) as the lowest and highest limits for the confident intervals (average 1.46). None of the other variants analysed were significantly associated with BMI after Bonferroni correction. The only SNPs, other than *FTO* which had a p-value lower than 0.05 for BMI, were the *rs17782313* close to the *MC4R* gene and the *rs10838738* within the *MTCH2* gene. When DM2 was included as co-variable the results did not change.

#### Hortega population

This study included 1502 subjects but 77 were excluded because of low genotyping rate (<95%). The average genotyping call rate after removing them was 99.7 ± 0.7%. As it was in Pizarra population only the *rs7561317* polymorphism had to be excluded. The average SNP call rate was 99.7 ± 0.5%.

In this population none of the SNPs or haplotypes reached the significance after Bonferroni correction (Table 3). The most strongly associated SNPs were on chromosome 1 near the neuronal growth regulator 1 precursor (*NEGR1*) gene and are in high LD (beta -0.40, p-value 0.008 for the association of *rs3101336* with BMI). Other SNPs around nominal p-values for significance were *rs8049439* located in the *ATXN2L* gene on chromosome 16 [beta 0.33 (0.02-0.63), p-value 0.03] and *rs3813929* within the HTR2C gene on chromosome X [beta -0.43 (-0.88-0.01), p-value 0.05]. The addition of DM2 did not affect the results of the individual SNPs analysis.

#### Pooled analyses

The pooled sample size included 2490 individuals but 196 were excluded because of low genotyping call rate (<95%). All the SNPs in high LD within the *FTO* gene (Additional file 1: Figure S1) were nominally associated with BMI, Table 3. The allele *A* of the *rs9939609* produced an increase of 0.29 in BMI [beta 0.29 (0.03-0.56), p-value 0.02]. In this case, the most associated SNP was close to de HTR2C gene on chromosome X. The allele *T* of this SNP produced a decrease of about 0.5 in BMI [beta -0.55 (-0.96 - -0.15), p-value 0.007].

Although a formal test for interaction did not reveal significant interaction between the SNPs, *rs6499640* and *rs1421085* of *FTO*, however the double homozygotes for the minor allele had significantly higher BMI values than double homozygotes for the wild allele (Additional file 2: Figure S2).

The information about the association of the non-Tag SNPs with BMI can be consulted in the Additional file 3: Table S1. Information about the LD and haploblocks can be consulted in the Additional file 1: Figure S1. The haplotype association analysis can be consulted in the Additional file 4: Table S2.

### Meta-analysis and statistical heterogeneity

For those SNPs in the FTO gene in high LD, the level of statistical heterogeneity based on the p-value for Cochrane’s Q statistic and the I2 heterogeneity index, was very high.

For the rest of the SNPs in other loci, the level of heterogeneity was low or moderate.

None of the SNPs reached the statistical significance after Bonferroni correction.

Only three SNPs were nominally associated with BMI in the meta-analysis, *rs9939609* of *FTO* (in the fixed effect model), *rs3101336* of the *NEGR1* gene *(*both fixed and random effects models) and *rs3813929* of the *HTR2C* gene (both fixed and random effects models)*.*

The results for the meta-analysis and the statistical heterogeneity are shown in Table 4.

**Table 4:** Meta-analysis for the association of BMI with individual SNPs, for Pizarra and Hortega studies, and level of statistical heterogeneity for each SNP

						**BMI**			
**CHR**	**Gene**	**SNP**	**A1**	**P**	**P(R)**	**Beta**	**Beta(R)**	**Q**	**I**
16	*FTO*	rs9939609	A	0.004768	0.1902	0.3544	0.4843	0.0071	86.22
		rs8044769	T	0.05001	0.1921	−0.245	−0.3108	0.0788	67.64
		rs7190492	A	0.8216	0.6838	−0.0299	−0.1556	0.0076	85.97
		rs6499640	G	0.1948	0.1948	0.1659	0.1659	0.3853	0
18	*MC4R*	rs17782313	C	0.1092	0.1872	0.2563	0.2941	0.1879	42.34
11	*MTCH2*	rs10838738	G	0.1222	0.1837	0.2031	0.2281	0.2157	34.75
1	*NEGR1*	rs3101336	A	0.0154	0.04555	−0.3124	−0.2989	0.2647	19.6
16	*ATXN2L*	rs8049439	C	0.1431	0.5759	0.1893	0.1295	0.0975	63.58
23	*HTR2C*	rs3813929	T	0.02603	0.02603	−0.4272	−0.4272	0.9414	0
5	*ADRB2*	rs12654778	A	0.8699	0.9013	−0.0208	0.0254	0.1347	55.32
1	*SEC16B*	rs10913469	C	0.9855	0.8286	−0.0031	0.0586	0.149	51.97
2	*TMEM18*	rs4854344	G	0.1106	0.1106	−0.2701	−0.2701	0.781	0
	*INSIG2*	rs7566605	C	0.9423	0.9423	0.0097	0.0097	0.652	0
3	*ADIPOQ*	rs17300539	A	0.758	0.758	0.0573	0.0573	0.6433	0
		rs3774261	A	0.6931	0.6931	−0.0494	−0.0494	0.9466	0
	*ETV5*	rs7647305	T	0.2549	0.2549	0.1763	0.1763	0.5226	0
4	*GNPDA2*	rs10938397	G	0.2632	0.2853	−0.1393	−0.1474	0.2784	14.87
10	*PTER*	rs10508503	T	0.2284	0.2284	−0.2387	−0.2387	0.7263	0
11	*BDNF*	rs925946	T	0.9293	0.9293	−0.012	−0.012	0.4992	0
		rs10501087	C	0.633	0.633	0.0701	0.0701	0.8626	0
12	*FAIM2*	rs7138803	A	0.9989	0.9989	−0.0002	−0.0002	0.4393	0
18	*NPC1*	rs1805081	G	0.9971	0.9971	−0.0005	−0.0005	0.9492	0
19	*KCTD15*	rs11084753	A	0.5295	0.5295	−0.0808	−0.0808	0.3724	0
20	*CTNNBL1*	rs6013029	T	0.7034	0.7034	−0.0914	−0.0914	0.738	0

*CHR*: Chromosome code; *SNP*: SNP identifier; *A1*: Minor allele code; *P*: Fixed-effects meta-analysis p-value; *P(R)*: Random-effects meta-analysis p-value; *BETA*: Fixed-effects BETA estimate; *BETA(R)*: Random-effects BETA estimate; *Q*: p-value for Cochrane’s Q statistic; *I*: I^2^heterogeneity index (0-100).

### Weighted genetic risk score

A genetic risk score was constructed with the risk alleles of the six tagSNPs with a positive Beta in Hortega, Pizarra and the Pooled analysis, that is: *rs9939609* and *rs6499640* of the *FTO* gene*; rs17782313* of the *MC4R* gene; *rs10838738* of the *MTCH2* gene; *rs7647305* of the *ETV5* gene; and *rs10501087* of the BDNF gene.

**Figure 1:**
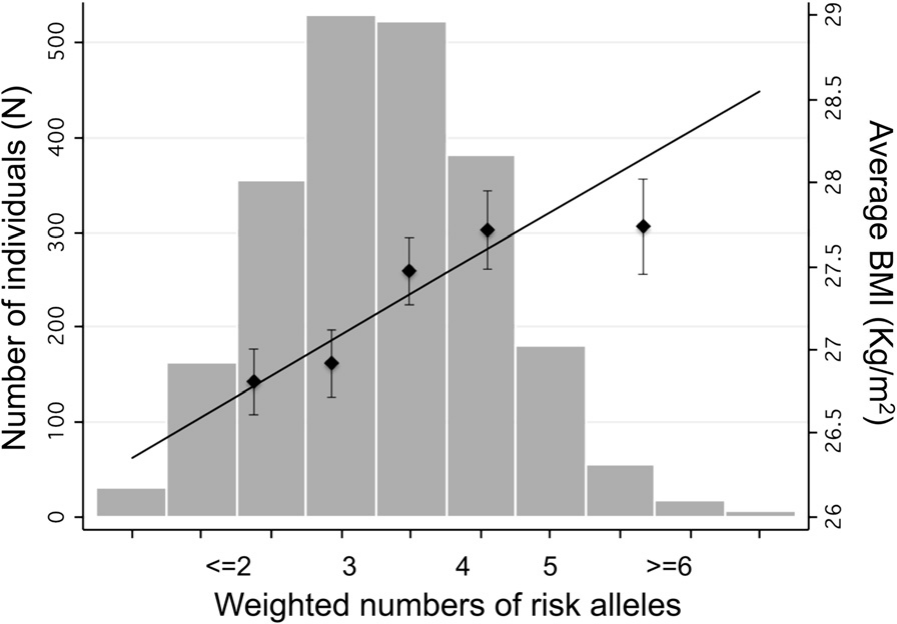
**Combined impact of risk alleles on average BMI in the pooled analysis.** For each individual, the number of risk alleles (0,1,2) per SNP was weighted for their effect sizes and re-scaled by dividing by the average of the all the effect sizes. The weighted risk alleles for the selected SNPs were summed for each individual, and the overall individual sum was rounded to the nearest integer to represent the individual’s risk allele score. Along the x axis, individuals in each risk allele category are shown (grouped ≤2 and ≥6 at the extremes), and the mean BMI (± s.e.m.) is plotted (y axis on right), with the line representing the regression of the mean BMI values across the risk allele scores. The histogram (y axis on left) represents the number of individuals for each risk-score category.

The score constructed with the sum of the weighed risk alleles was positively correlated with BMI in Pizarra and in the pooled sample (r = 0.15 in Pizarra, p-value < 0.001; r = 0.082 in the pooled sample, p-value < 0.001). From the first to the fifth quintile of the score, the BMI increases 2 Kg/m^2^ in Pizarra, 0.45 Kg/m^2^ in Hortega and 0.93 Kg/m^2^ in the pooled analysis. The BMI values for each quintile of the score as well as the regression line between BMI and the risk score is shown in Figure 1. However, the variance of BMI associated to individual SNPs or to the score was very low (less than 1%). The predictive value of the score for obesity was poor [area under the curve (AUC) 0.515 and 0.594 in Hortega and Pizarra respectively] (Additional file 5: Figure S3).

## Discussion

The present study, carried out in two Spanish populations with different characteristics, confirms the association of BMI with some of the genes previously described and provides further evidence of the influence of the population characteristics on the association level. Variants in the *FTO* gene were significantly associated with BMI in one of the populations, Pizarra, whereas none of the selected variants were significantly associated in Hortega population or in the pooled analysis.

A large battery of previously described genes associated with BMI and obesity were selected for the present study. They included not only the best characterized *FTO* gene but also others recently described for which the association was not as strong or was less consistently reproduced. Among them, *FTO*, *MC4R*, *MTCH2* and *HTR2C* are the main associated loci with BMI and obesity. Among the new discovered loci, *ATXN2L*, *NEGR1* and *SH2B1* have also shown a relationship with obesity.

Virtually all the SNPs in LD in the intron 1 of *FTO* were associated with BMI in Pizarra population. Furthermore, another SNP of *FTO*, *rs6499640*, not in LD with the others was also associated with BMI when it was included in the haplotypes. The estimated effect of the minor allele of these SNPs was very low in concordance with published studies in which carriers of the minor allele of *rs9939609* weighed about three kg more than carriers of the other allele [4]. In the Pizarra population, the strongest association was with *rs9939609* although the majority of polymorphisms in high LD with it reached the significance even after adjusting by confounding factors and by multiple comparisons. In the meta-analysis performed by Willer et al [7], the estimated effect for these variants ranged from 0.06 to 0.33 kg/m^2^, what means a change in weight between 173-954 g per allele for adults who are 1.60-1.80 m height [7]. The estimated maximum effect of *FTO* gene alleles was higher in Pizarra population as compare to the results of the meta-analysis although in the pooled analysis the overall effect was similar.

In contrast with the strong association of *FTO* in the Pizarra population, there was a weak association in Hortega population. This difference could be attributed to the characteristics of the two populations. The Pizarra population was younger and had significantly higher mean BMI and prevalence of obesity than Hortega population. In fact, it has been suggested that the contribution of *FTO* may be more evident in very obese and younger populations [19]. There is also a trend toward less association with increase in age in some studies [4,20].

As a consequence of the low estimated effect of the variants, none of the other loci out of *FTO* reached the statistical significance after multiple comparisons adjustments. However, some of them which were close to the nominal p-value merit some comments. After *FTO*, the most associated SNP with BMI in Pizarra population was the *rs17782313* of *MC4R* which it is in agreement with previous meta-analysis [7,21]. Located 188 kb downstream of the *MC4R*, it was first identified using GWAS in 16876 individuals of European descent and replicated in 60352 adults. *MC4R* is a strong candidate gene for obesity because functional mutations of this gene are associated with monogenic forms of obesity [22]. The reported effect associated with this variant was lower than the effect reported for the *FTO* variants. The power to detect a true association decreases when the associated effect of that variant decrease.

The integration of the information of relevant SNPs into a genetic risk score might be a way to select those subjects at high risk to develop obesity in the future. By using the information provided in the present study, a weighted genetic risk score (wGRS) was constructed by using those Tag-SNPs with a positive Beta in Hortega, Pizarra and the pooled analysis. This wGRS include the following SNPs: *rs9939609* and *rs6499640* of the *FTO* gene*; rs17782313* of the *MC4R* gene; *rs10838738* of the *MTCH2* gene; *rs7647305* of the *ETV5* gene; and *rs10501087* of the *BDNF* gene.

From the first to the fifth quintile of the score, the BMI increased 0.45 kg/m^2^ in Hortega, 2.00 kg/m^2^ in Pizarra and 0.94 kg/m^2^ in the pooled analysis. These data are in agreement to those obtained by the Genetic Investigation of Anthropometric Traits (GIANT) project in which subjects with the highest score weighed on average 1.46 kg/m^2^ more than those with the lowest score [7].

Despite the significant differences observed in BMI and in the prevalence of obesity according to the score categories, the explained variance for BMI was less than 1%, similar to the one predicted in a previous study for Willer and colleagues [7]. Several potential explanations can be offered for the low predictive value of the wGRS but are mainly related with the marginal effect sizes of the tested variants and the skewed distribution of the effect sizes [23]. Other potential explanations for the low predictive value of the wGRS could be related with the gene-gene or especially with the gene-environment interactions which were not considered in the present study [23]. Because the majority of these genes are expressed in the central nervous system, acting in appetite regulation, behaviour and basal energy expenditure [7,24-26], the importance of environmental factors, mainly high energy intake and low physical activity, should be considered. Estimation of energy intake and physical activity, however, are unreliable because of under- or over-reported [27]. Since the effect of these genetic variants might be due to an increase of energy intake [28], inclusion of energy intake could lead to a marked improvement in prediction. This could have clinical impact if we are able to identify those individuals in which energy restriction below some threshold should be strongly recommended.

The main limitation of the present study is that our sample size was too small to detect association for the majority of the tested variants with low estimated effect, although we pooled two different populations to overcome this problem. This may have influenced the strength of the association but not the size of the effect, which was similar to that reported in one meta-analysis [7]. The appropriateness of combining these two populations in one is somehow controversial and merits some comments. Both samples share similar genetic backgrounds due to the low immigration rate and are supposed to be homogeneous ethnic populations. Because of this, we did not expect population stratification in our samples what can favour the pooled strategy. However, they belong to different geographical regions of Spain and they have completely different clinical characteristics. This clinical heterogeneity probably has had a great influence in the pooled analysis results. For this reason we also decided to perform a meta-analysis to assess the statistical heterogeneity. For some of the SNPs such as those of the *FTO* gene, the level of statistical heterogeneity based on the p-value for Cochrane’s Q statistic and the I2 heterogeneity index, was very high. For other SNPs, such as the *rs12654778* at *ADRB2* with a priori adequate statistical power to detect significant association, we did not find any. The clinical heterogeneity and the level of statistical heterogeneity, at least moderate, can justify the lack of association. Besides we cannot be sure for certain that the prior statistical power for that SNP is that high due to the previously commented considerations.

The different lipid and carbohydrate metabolic profiles observed between populations may be related with the different characteristics of the target populations with markedly different lifestyles. Several studies have shown that the effects of *FTO* alleles are attenuated by exercise [29,30]. Individuals from Pizarra population belonged to a rural area and were significantly younger than those from the Hortega study. The individuals from the latter study were recruited in the area covered by a tertiary hospital and the majority of them lived in urban areas. This population was also even regarding to gender distribution as compare with Pizarra population, which included mostly females. Because of the potential influence of the population to which each individual belongs, we also adjusted the analysis by this factor, and the results remained unchanged.

## Conclusions

In conclusion, baseline characteristics of the populations, mainly age and grade of obesity, have a strong influence in the genetic association results. *FTO was* the only locus that was clearly associated with BMI in this study. None of the other loci including the *MC4R*, *MTCH2* or the newly discovered ones, such as *ATXN2L*, *NEGR1* and *SH2B1* were associated with BMI in this study. The risk associated with these polymorphisms is low and the overall effect in BMI is minimal. Considering the high heritability of obesity, new variants remain to be discovered. As commented previously, the majority of the analysed loci are related to central nervous system mechanisms of obesity but many other mechanisms can influence body weight and their contribution have not been elucidated yet. New strategies, like the study of lean individuals [31-34], translational information from animal models [35-38], nutrigenomics [39,40], as well as the interaction with energy intake and physical activity may lead to a better understanding of the genetic component in the physiopathology of obesity.

## Competing interests

We declare that we have not received reimbursements, fees, funding, or salary from organizations that may in any way gain or lose financially from the publication of this manuscript, either now or in the future.

We declare that we do not hold any stocks or shares in organizations that may in any way gain or lose financially from the publication of this manuscript, either now or in the future.

We declare that we do not hold and that we are not applying for any patents relating to the content of the manuscript.

We declare that we have not received reimbursements, fees, funding, or salary from an organization that holds or has applied for patents relating to the content of the manuscript.

We declare that we do not have any other financial or non-financial competing interests.

## Authors’ contributions

FM: Develop the main analysis, wrote and edited the manuscript. MLM, DdM and FJC: Designed the study, made the genotyping and edited the manuscript. JR: Designed the study and reviewed the manuscript. GR; SM; and FS are the main investigators of the Pizarra study. Edited and reviewed the article. MP and JCE, are the main investigator of the Hortega study. Edited and reviewed the article. RSC: Reviewed and edited the article. All authors read and approved the final manuscript.

## Supplementary Material

Additional file 1: Figure S1 Information about the LD and haploblocks for the following genes: *NEGR1*, *TMEM18*, *ADIPOQ*, *BDNF*, *MTCH2* and *FTO.* Click here for file

Additional file 2: Figure S2 Mean of BMI according to genotypes of *rs1421085* and *rs6499640* of *FTO* adjusted by age and gender in the pooled sample. Click here for file

Additional file 3: Table S1 Individual association for the NonTag-SNPs with BMI adjusted by age and gender under an additive genetic model in Pizarra, Hortega and the pooled analysis. Click here for file

Additional file 4: Table S2 Haplotype association analysis with BMI adjusted by age and gender in Pizarra, Hortega and in the pooled analysis. Click here for file

Additional file 5: Figure S3 Areas under the curve for the obesity prediction for the weighted genetic risk score in Pizarra and Hortega populations. Click here for file
