# Supplementary figures and images for: Impact of obesity-related genes in Spanish population
Source: BMC Genet. 2013 Nov 23;14:111. doi: 10.1186/1471-2156-14-111 (PMC4222487; doi:10.1186/1471-2156-14-111)

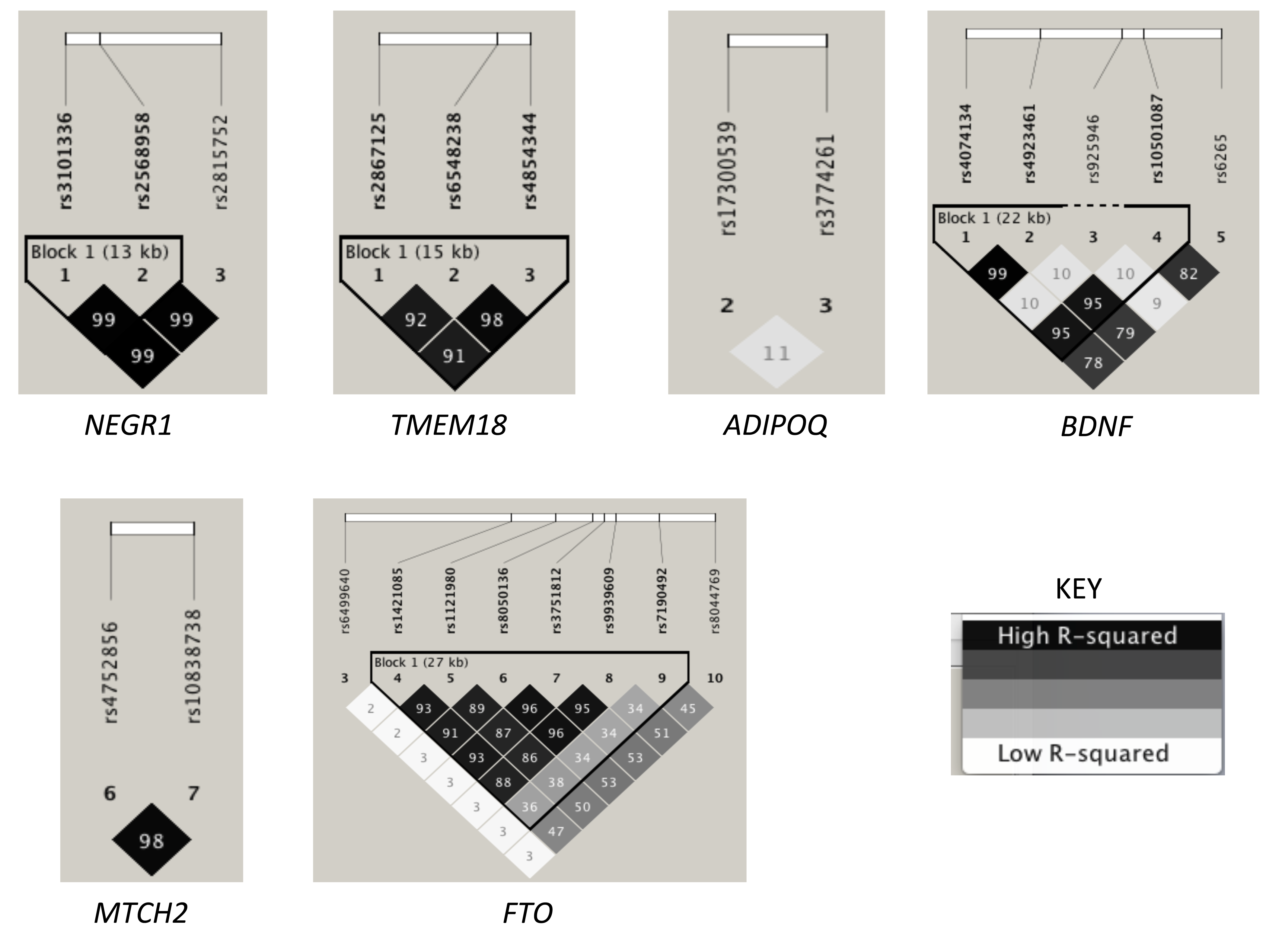

Supplement: Additional file 1: Figure S1 — Information about the LD and haploblocks for the following genes: NEGR1, TMEM18, ADIPOQ, BDNF, MTCH2 and FTO. [file 1471-2156-14-111-S1.tiff]

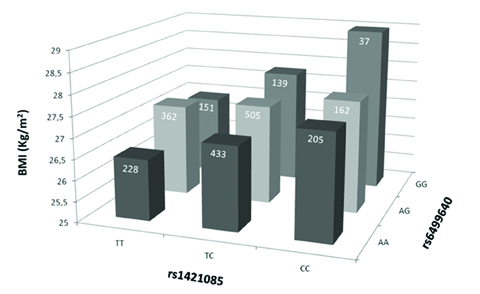

Supplement: Additional file 2: Figure S2 — Mean of BMI according to genotypes of rs1421085 and rs6499640 of FTO adjusted by age and gender in the pooled sample. [file 1471-2156-14-111-S2.tiff]

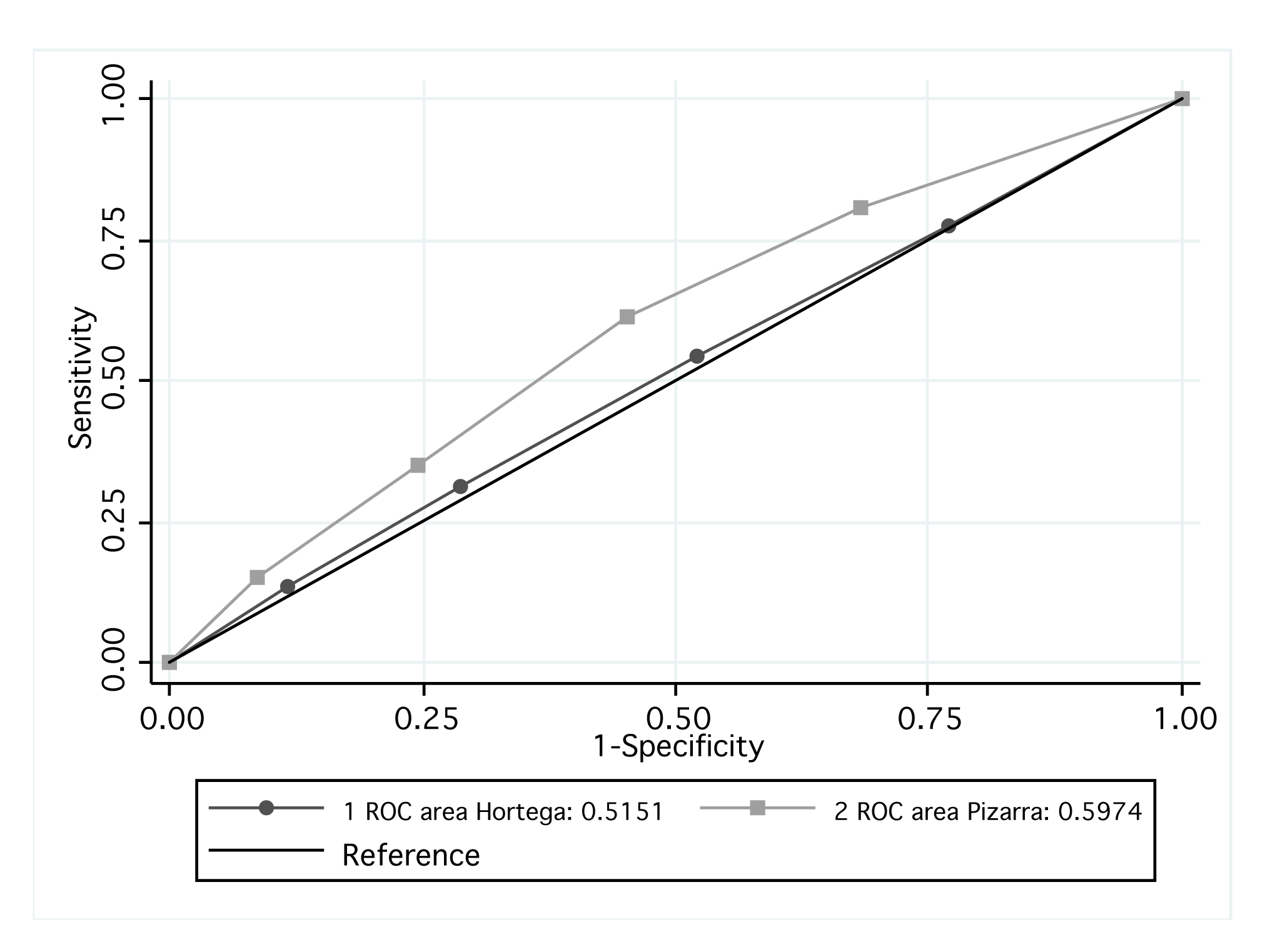

Supplement: Additional file 5: Figure S3 — Areas under the curve for the obesity prediction for the weighted genetic risk score in Pizarra and Hortega populations. [file 1471-2156-14-111-S5.tiff]
