# Supplementary material for: Impact of obesity-related genes in Spanish population
Source: BMC Genet. 2013 Nov 23;14:111. doi: 10.1186/1471-2156-14-111 (PMC4222487; doi:10.1186/1471-2156-14-111)
Supplement: Additional file 3: Table S1 — Individual association for the NonTag-SNPs with BMI adjusted by age and gender under an additive genetic model in Pizarra, Hortega and the pooled analysis. [file 1471-2156-14-111-S3.docx]

***Additional file 3: Table S1.*** Individual SNP (non tag-SNPs) association analysis with BMI adjusted by age and gender under an additive genetic model in Pizarra, Hortega and the pooled analysis.

|  |  |  |  |  |  | **BMI** | | | | |
| --- | --- | --- | --- | --- | --- | --- | --- | --- | --- | --- |
|  | **GENE** | **SNP** | **A1** | **MAF** | **POPULATION** | **N*** | **BETA** | **STANDARD**  **ERROR** | **STAT**** | **P** |
|  | ***FTO*** | ***rs8050136*** | ***A*** | **0.3898** | **PIZARRA** | **841** | **0.887** | **0.2353** | **3.76** | **0.0001752** |
|  |  |  |  | 0.4311 | HORTEGA | 1347 | 0.1747 | 0.1513 | 1.15 | 0.249 |
|  |  |  |  | 0.4147 | POOLED | 2210 | 0.3105 | 0.1378 | 2.25 | 0.0244 |
|  |  | ***rs1121980*** | ***T*** | **0.4206** | **PIZARRA** | **848** | **0.822** | **0.2296** | **3.58** | **0.0003637** |
|  |  |  |  | 0.4515 | HORTEGA | 1348 | 0.2171 | 0.1501 | 1.44 | 0.148 |
|  |  |  |  | 0.4392 | POOLED | 2218 | 0.3484 | 0.1359 | 2.56 | 0.0104 |
|  |  | ***rs1421085*** | ***C*** | **0.4039** | **PIZARRA** | **848** | **0.802** | **0.2305** | **3.48** | **0.0005241** |
|  |  |  |  | 0.4347 | HORTEGA | 1348 | 0.2754 | 0.1496 | 1.84 | 0.0659 |
|  |  |  |  | 0.4226 | POOLED | 2218 | 0.3686 | 0.1358 | 2.71 | 0.0067 |
|  |  | ***rs3751812*** | ***T*** | **0.3849** | **PIZARRA** | **848** | **0.772** | **0.2348** | **3.28** | **0.001053** |
|  |  |  |  | 0.4269 | HORTEGA | 1348 | 0.1922 | 0.1503 | 1.27 | 0.201 |
|  |  |  |  | 0.4102 | POOLED | 2218 | 0.2741 | 0.1371 | 1.99 | 0.0457 |
|  | *MTCH2* | *rs4752856* | *A* | 0.3472 | PIZARRA | 846 | 0.455 | 0.2411 | 1.88 | 0.0592 |
|  |  |  |  | 0.3513 | HORTEGA | 1348 | 0.1049 | 0.1567 | 0.66 | 0.503 |
|  |  |  |  | 0.3501 | POOLED | 2216 | 0.1646 | 0.142 | 1.15 | 0.2467 |
|  | *NEGR1* | *rs2815752* | *C* | 0.3751 | PIZARRA | 848 | -0.084 | 0.2418 | -0.34 | 0.7277 |
|  |  |  |  | 0.3434 | HORTEGA | 1348 | -0.403 | 0.1524 | -2.64 | 0.0083 |
|  |  |  |  | 0.3555 | POOLED | 2218 | -0.1622 | 0.1395 | -1.16 | 0.2452 |
|  | *NEGR1* | *rs2568958* | *G* | 0.3751 | PIZARRA | 848 | -0.084 | 0.2418 | -0.34 | 0.7277 |
|  |  |  |  | 0.3442 | HORTEGA | 1348 | -0.3792 | 0.152 | -2.49 | 0.0127 |
|  |  |  |  | 0.3559 | POOLED | 2218 | -0.1489 | 0.1393 | -1.06 | 0.2852 |
|  | *SH2B1* | *rs4788102* | *A* | 0.3558 | PIZARRA | 846 | -0.155 | 0.2396 | -0.64 | 0.5167 |
|  |  |  |  | 0.327 | HORTEGA | 1348 | 0.2902 | 0.1571 | 1.84 | 0.0649 |
|  |  |  |  | 0.3388 | POOLED | 2216 | 0.1323 | 0.1416 | 0.93 | 0.3501 |
|  | *TMEM18* | *rs6548238* | *T* | 0.1694 | PIZARRA | 847 | -0.347 | 0.3092 | -1.12 | 0.2611 |
|  |  |  |  | 0.1698 | HORTEGA | 1348 | -0.2927 | 0.2028 | -1.44 | 0.1493 |
|  |  |  |  | 0.1699 | POOLED | 2217 | -0.3181 | 0.1823 | -1.74 | 0.0811 |
|  | *TMEM18* | *rs2867125* | *A* | 0.172 | PIZARRA | 848 | -0.250 | 0.3075 | -0.81 | 0.4159 |
|  |  |  |  | 0.1681 | HORTEGA | 1344 | -0.2854 | 0.2041 | -1.39 | 0.1621 |
|  |  |  |  | 0.1699 | POOLED | 2211 | -0.2681 | 0.1829 | -1.46 | 0.1428 |
|  | *BDNF* | *rs6265* | *A* | 0.2284 | PIZARRA | 848 | 0.037 | 0.2669 | 0.13 | 0.89 |
|  |  |  |  | 0.2166 | HORTEGA | 1346 | 0.113 | 0.1816 | 0.62 | 0.5339 |
|  |  |  |  | 0.2212 | POOLED | 2215 | 0.0810 | 0.1609 | 0.50 | 0.6145 |
|  | *BDNF* | *rs4923461* | *G* | 0.2598 | PIZARRA | 847 | -0.004 | 0.2573 | -0.01 | 0.9863 |
|  |  |  |  | 0.2343 | HORTEGA | 1348 | 0.1244 | 0.1777 | 0.7 | 0.4841 |
|  |  |  |  | 0.2441 | POOLED | 2216 | 0.1566 | 0.1564 | 1.01 | 0.3169 |
|  | *BDNFOS* | *rs4074134* | *A* | 0.2618 | PIZARRA | 844 | -0.013 | 0.2569 | -0.05 | 0.9573 |
|  |  |  |  | 0.235 | HORTEGA | 1348 | 0.1295 | 0.1769 | 0.73 | 0.4644 |
|  |  |  |  | 0.2454 | POOLED | 2214 | 0.1590 | 0.1559 | 1.02 | 0.3079 |

A1: minor allele, *Number of non-missing individuals included in the analysis;** t statistic coefficient; p- values are not corrected for multiple testing; Bold type indicates significant association after Bonferroni correction.
