# Supplementary material for: Impact of obesity-related genes in Spanish population
Source: BMC Genet. 2013 Nov 23;14:111. doi: 10.1186/1471-2156-14-111 (PMC4222487; doi:10.1186/1471-2156-14-111)
Supplement: Additional file 4: Table S2 — Haplotype association analysis with BMI adjusted by age and gender in Pizarra, Hortega and in the pooled analysis. [file 1471-2156-14-111-S4.docx]

***Additional file 4: Table S2.*** Haplotype association analysis with BMI adjusted by age and gender in Pizarra, Hortega and in the pooled analysis.

|  |  |  | |  | **POPULATION** | **BMI** | | | |
| --- | --- | --- | --- | --- | --- | --- | --- | --- | --- |
|  | **GENE** | **SNP** | | **A1** |  | **N*** | **BETA** | **STAT**** | **P** |
| Haplotype analysis | **FTO** | **rs9939609** | **rs7190492** | **AG** | **PIZARRA** | **0.394** | **0.902** | **15.1** | **0.00011** |
|  |  |  |  |  | HORTEGA | 0.432 | 0.145 | 0.934 | 0.334 |
|  |  |  |  |  | POOLED | 0.417 | 0.312 | 5.22 | 0.0225 |
|  |  | **rs3751812** | **rs9939609** | **GT** | PIZARRA | **0.601** | **-0.875** | **14.4** | **0.00016** |
|  |  |  |  |  | HORTEGA | 0.564 | -0.135 | 0.811 | 0.368 |
|  |  |  |  |  | POOLED | 0.579 | -0.298 | 4.79 | 0.0287 |
|  |  | **rs1121980** | **rs8050136** | **TA** | **PIZARRA** | **0.389** | **0.882** | **14.1** | **0.00019** |
|  |  |  |  |  | HORTEGA | 0.43 | 0.178 | 1.38 | 0.24 |
|  |  |  |  |  | POOLED | 0.414 | 0.314 | 5.21 | 0.0226 |
|  |  | **rs8050136** | **rs3751812** | **CG** | **PIZARRA** | **0.609** | **-0.874** | **13.8** | **0.00021** |
|  |  |  |  |  | HORTEGA | 0.566 | -0.191 | 1.61 | 0.205 |
|  |  |  |  |  | POOLED | 0.583 | -0.315 | 5.25 | 0.022 |
|  |  | **rs1121980** | **rs8050136** | **CC** | **PIZARRA** | **0.579** | **-0.851** | **13.6** | **0.00024** |
|  |  |  |  |  | HORTEGA | 0.547 | -0.216 | 2.06 | 0.152 |
|  |  |  |  |  | POOLED | 0.56 | -0.353 | 6.69 | 0.00976 |
|  |  | **rs1421085** | **rs1121980** | **TC** | **PIZARRA** | **0.578** | **-0.810** | **12.5** | **0.00044** |
|  |  |  |  |  | HORTEGA | 0.549 | -0.217 | 2.09 | 0.148 |
|  |  |  |  |  | POOLED | 0.56 | -0.349 | 6.59 | 0.0103 |
|  |  | **rs1421085** | **rs1121980** | **CT** | **PIZARRA** | **0.403** | **0.814** | **12.5** | **0.00044** |
|  |  |  |  |  | HORTEGA | 0.435 | 0.275 | 3.39 | 0.066 |
|  |  |  |  |  | POOLED | 0.422 | 0.368 | 7.36 | 0.00674 |
|  |  | **rs6499640** | **rs1421085** | **AT** | **PIZARRA** | **0.349** | **-0.895** | **12.3** | **0.00047** |
|  |  |  |  |  | HORTEGA | 0.300 | -0.341 | 3.90 | 0.049 |
|  |  |  |  |  | POOLED | 0.32 | -0.45 | 8.58 | 0.00344 |
|  |  | **rs8050136** | **rs3751812** | **AT** | **PIZARRA** | **0.385** | **0.796** | **11.4** | **0.00078** |
|  |  |  |  |  | HORTEGA | 0.425 | 0.177 | 1.38 | 0.241 |
|  |  |  |  |  | POOLED | 0.409 | 0.277 | 4.03 | 0.0448 |
|  |  | **rs3751812** | **rs9939609** | **TA** | **PIZARRA** | **0.385** | **0.772** | **10.8** | **0.00105** |
|  |  |  |  |  | HORTEGA | 0.426 | 0.192 | 1.62 | 0.203 |
|  |  |  |  |  | POOLED | 0.41 | 0.275 | 4.02 | 0.0451 |
|  |  | **rs6499640** | **rs1421085** | **GC** | PIZARRA | 0.129 | 1.080 | 8.09 | 0.0046 |
|  |  |  |  |  | HORTEGA | 0.116 | 0.334 | 1.61 | 0.204 |
|  |  |  |  |  | POOLED | 0.122 | 0.714 | 9.43 | 0.00216 |
|  |  | rs9939609 | rs7190492 | TG | PIZARRA | 0.277 | -0.455 | 3.03 | 0.0822 |
|  |  |  |  |  | HORTEGA | 0.230 | -0.473 | 6.92 | 0.0086 |
|  |  |  |  |  | POOLED | 0.249 | -0.326 | 4.26 | 0.0392 |
|  |  | rs9939609 | rs7190492 | TA | PIZARRA | 0.323 | -0.546 | 5.18 | 0.0231 |
|  |  |  |  |  | HORTEGA | 0.334 | 0.218 | 1.85 | 0.174 |
|  |  |  |  |  | POOLED | 0.33 | -0.0647 | 0.203 | 0.653 |
|  |  | rs7190492 | rs8044769 | AT | PIZARRA | 0.306 | -0.474 | 3.64 | 0.0566 |
|  |  |  |  |  | HORTEGA | 0.317 | 0.149 | 0.83 | 0.363 |
|  |  |  |  |  | POOLED | 0.313 | -0.0861 | 0.341 | 0.559 |
|  |  | rs1121980 | rs8050136 | TC | PIZARRA | 0.031 | -0.018 | 0.00071 | 0.979 |
|  |  |  |  |  | HORTEGA | 0.022 | 0.508 | 0.97 | 0.325 |
|  |  |  |  |  | POOLED | 0.0256 | 0.476 | 1.24 | 0.265 |
|  | *NEGR1* | rs3101336 | rs2568958 | AG | PIZARRA | 0.375 | -0.084 | 0.121 | 0.728 |
|  |  |  |  |  | HORTEGA | 0.343 | -0.400 | 6.88 | 0.0089 |
|  |  |  |  |  | POOLED | 0.355 | -0.152 | 1.19 | 0.275 |
|  |  | rs2568958 | rs2815752 | GC | PIZARRA | 0.375 | -0.084 | 0.121 | 0.728 |
|  |  |  |  |  | HORTEGA | 0.343 | -0.400 | 6.88 | 0.0089 |
|  |  |  |  |  | POOLED | 0.355 | -0.159 | 1.29 | 0.256 |
|  | *BDNF* | rs10501087 | rs6265 | CG | PIZARRA | 0.031 | -0.394 | 0.331 | 0.565 |
|  |  |  |  |  | HORTEGA | 0.023 | -0.256 | 0.27 | 0.606 |
|  |  |  |  |  | POOLED | 0.026 | 0.22 | 0.263 | 0.608 |

A1: minor allele, * Frequency of the haplotype in the sample;** t statistic coefficient; p- values are not corrected for multiple testing; Bold type indicates significant association after Bonferroni correction.
